# SPIN1, negatively regulated by miR-148/152, enhances Adriamycin resistance via upregulating drug metabolizing enzymes and transporter in breast cancer 

**DOI:** 10.1186/s13046-018-0748-9

**Published:** 2018-05-09

**Authors:** Xu Chen, Ya-Wen Wang, Peng Gao

**Affiliations:** Department of Pathology, School of Medicine, Shandong University, 44 Wen Hua Xi Road, Jinan, 250012 People’s Republic of China

**Keywords:** Breast cancer, Adriamycin resistance, SPIN1, miR-148/152, Drug metabolizing enzymes, Drug transporter

## Abstract

**Background:**

Spindlin1 (SPIN1), a protein highly expressed in several human cancers, has been correlated with tumorigenesis and development. Alterations of drug metabolizing enzymes and drug transporters are major determinants of chemoresistance in tumor cells. However, whether the metabolizing enzymes and transporters are under the control of SPIN1 in breast cancer chemoresistance has not yet been defined.

**Methods:**

SPIN1 expression in breast cancer cells and tissues was detected by quantitative real-time PCR (qRT-PCR) and immunohistochemistry. Chemosensitivity assays in vitro and in vivo were performed to determine the effect of SPIN1 on Adriamycin resistance. Downstream effectors of SPIN1 were screened by microarray and confirmed by qRT-PCR and Western blot. Luciferase assay and Western blot were used to identify miRNAs regulating *SPIN1*.

**Results:**

We showed that SPIN1 was significantly elevated in drug-resistant breast cancer cell lines and tissues, compared with the chemosensitive ones. SPIN1 enhanced Adriamycin resistance of breast cancer cells in vitro*,* and downregulation of SPIN1 by miRNA could decrease Adriamycin resistance in vivo. Mechanistically, drug metabolizing enzymes and transporter CYP2C8, UGT2B4, UGT2B17 and ABCB4 were proven to be downstream effectors of SPIN1. Notably, *SPIN1* was identified as a direct target of the miR-148/152 family (miR-148a-3p, miR-148b-3p and miR-152-3p). As expected, miR-148a-3p, miR-148b-3p or miR-152-3p could increase Adriamycin sensitivity in breast cancer cells in vitro. Moreover, high expression of *SPIN1* or low expression of the miR-148/152 family predicted poorer survival in breast cancer patients.

**Conclusions:**

Our results establish that SPIN1, negatively regulated by the miR-148/152 family, enhances Adriamycin resistance in breast cancer via upregulating the expression of drug metabolizing enzymes and drug transporter.

**Electronic supplementary material:**

The online version of this article (10.1186/s13046-018-0748-9) contains supplementary material, which is available to authorized users.

## Background

Chemoresistance is a major obstacle for effective breast cancer chemotherapy [1]. The generation or acquisition of cancer chemoresistance is not clear understood but has been attributed to alterations in many molecular pathways, which include drug metabolizing enzymes and drug transporter genes [2]. Alterations in the efflux transporters of the ATP-binding cassette (ABC) family have been identified as major determinants of chemoresistance in tumor cells by decreasing the intracellular accumulation of drugs [3]. In addition, drug-metabolizing enzymes cytochrome P450 (CYP) and UDP-glucuronosyltransferases (UGTs) have also been reported to constitute a resistance phenotype [4–6]. So far, little is known about the regulation of efflux transporters and metabolizing enzymes, though it has been found to be governed by nuclear receptors [6].

*Spindlin (SPIN)*, a member of the *SPIN/SSTY* family, was first reported as a major maternal transcript expressed in the mouse during the transition from oocyte to embryo [7]. Recent evidences showed that human Spindlin1 (SPIN1) was highly expressed in ovarian cancer and liposarcoma, and may be implicated in tumorigenesis and development [8–10]. SPIN1 has been shown to be elevated in chemoresistant and metastatic breast cancer tissues and involved in PI3K/Akt-mediated chemoresistance [11]. SPIN1 has been shown to be a histone code reader to bind histone H3 trimethylated at lysine 4 (H3K4me3) [12, 13], a chromatin mark typically located at promoters and associated with active or poised genes [14]. However, whether SPIN1 could regulate the drug metabolizing enzymes and transporters has not been defined. Moreover, we have previously found that SPIN1 was directly regulated by microRNA-489 [11]. Most microRNAs (miRNAs) only modestly affect their mRNA targets in a fine-tuning manner [15], and it has been confirmed that multiple miRNAs could target the same gene, suggesting that it is the combination of all these activities that exerts huge impacts on the expression of miRNA target genes [16]. We hypothesize that SPIN1 may also be regulated by other miRNAs.

Here we show that SPIN1 is highly expressed in drug-resistant breast cancer cells and tissues. We find that SPIN1 increases breast cancer Adriamycin resistance via enhancing the expression of drug metabolizing enzymes and transporter CYP2C8, UGT2B4, UGT2B17 and ABCB4. Mechanistically, the miR-148/152 family could directly target SPIN1 and increase Adriamycin sensitivity in breast cancer cells.

## Methods

### Tissue samples

We enrolled 78 cases of breast cancer patients before their first neoadjuvant Adriamycin-based chemotherapy cycle, in Qilu Hospital of Shandong University (Jinan, China) between Jul 2008 and Feb 2014. The cases included in this study were invasive breast carcinomas (invasive ductal carcinoma, *n* = 73; invasive lobular carcinoma, *n* = 5). Pre-chemotherapy needle biopsy samples and the paired surgically removed samples were collected, and the patients were divided into a drug-resistant group and a drug-sensitive group according to the pathological Miller/Payne assessment [17]. This is a five-point scale that focuses on the principal manifestation of chemotherapeutic effect on reduction in tumor cellularity of resection samples compared with pre-treatment needle biopsy tissues. The tumors were scored blindly by two pathologists and agreement by consensus was achieved if necessary. Patients were divided into a drug-resistant group (Miller/Payne grades 1–2) and a drug-sensitive group (grades 3–5) using the Miller/Payne grading system, as previously described [18, 19]. This study was approved by the Ethics Committee of School of Medicine, Shandong University (approval code: 2012028).

### Immunohistochemistry (IHC)

The streptavidin-peroxidase-biotin (SP) immunohistochemical method was utilized to determine the expression of SPIN1 in breast cancer tissues and xenograft tumors. The paraffin-embedded specimens (4 μm) sections were incubated with the anti-SPIN1 antibody (1:150, Proteintech, 19531-1-AP). For negative controls, the anti-SPIN1 antibody was replaced with PBS. For each sample, 500 cells from five randomly chosen fields were counted. The samples were divided into SPIN1 low-expression (< 50% positivity) and high-expression (≥50%) groups, as previously described [11]. All slides were scanned and photographed in a Pannoramic P250 scanner (3DHistech, Hungary).

### Cell culture and transfection

Human breast cancer cell lines MCF-7, MDA-MB-231, and MDA-MB-468 were obtained from the American Type Culture Collection and cultured in Dulbecco’s modified Eagle’s medium (MCF-7) or Leibovitz’s L15 medium (MDA-MB-231 and MDA-MB-468), supplemented with 10% fetal bovine serum (FBS; Gibco BRL, Grand Island, NY, U.S.). MCF-7/ADM cells were derived by treating MCF-7 cells with stepwise increasing concentrations of Adriamycin over 8 months and cultured in RPMI-1640 medium supplemented with 10% FBS. To maintain their resistance, MCF-7/ADM cells were cultured in the presence of a low concentration of Adriamycin and passaged for 1 week in drug-free medium before the experiments. The identities of the cell lines were confirmed by STR profiling in 2017 by Guangdong Hybribio Biotech Ltd. (Guangzhou, China; http://www.hybribio.cn).

Cells were transfected with miRNA mimics (GenePharma, Shanghai, China), *SPIN1* siRNA (si-*SPIN1*, RiboBio, Guangzhou, China) or the respective negative controls (NC), using X-tremeGENE transfection reagent (Roche, Indianapolis, IN, U.S.). The entire SPIN1 coding sequences were cloned into the expression plasmid pcDNA3.1(+) (pcDNA3.1(+)-*SPIN1*) and the empty plasmid pcDNA3.1(+) was used as a control. Plasmids were transfected with Lipofectamine 2000 (Invitrogen, USA) according to the manufacturer’s instructions.

### Chemosensitivity assay

For cell chemosensitivity assay, cells (5 × 10^3^) were seeded in 96-well plates. Adriamycin (Dalian Meilun, China) was added to determine the sensitivity to chemotherapy. Cell viability was detected after 48h as previously described [11].

### Microarray analysis

MCF-7/ADM cells were treated with *SPIN1* siRNA or negative control for 48h, followed by RNA preparation, labeling and hybridization in NimbleGen Hybridization System. Signal intensity was calculated from digitized images captured by Axon GenePix 4000B scanner. KEGG pathway enrichment analysis was performed using the DAVID online tool. The microarray data were deposited in the Gene Expression Omnibus (GEO) database (accession number: GSE71141).

### RNA isolation and quantitative real-time PCR (qRT-PCR)

For mRNA quantitative analysis, total RNAs were prepared using Trizol (Invitrogen) and were reverse transcribed with a Rever Tra Ace qPCR RT Kit (Toyobo, Osaka, Japan). Quantitative real-time PCR (qRT-PCR) was performed in a total volume of 10-μl SYBR Green Real-time PCR Master Mix (Roche, Mannheim, Germany) on a Bio-Rad CFX™ 96 C1000 Real-Time system. For quantitative detection of miRNA, reverse transcription and qPCR assay were performed using the All-in-One™ miRNA qRT-PCR detection kit (Genecopeia, Rockville, MD, USA). Primers for miR-148a-3p (HmiRQP0204), miR-148b-3p (HmiRQP0206), miR-152-3p (HmiRQP0213) and U6 (RNU6B, HmiRQP9001; reference gene) were from GeneCopoeia.

### Luciferase assay

The pmirGLO vector (Promega) was used to construct the recombinant plasmid pmirGLO-*SPIN1* containing the *SPIN1* mRNA 3’-UTR fragments which possess binding sites of miR-148a-3p, miR-148b-3p and miR-152-3p [11]. The luciferase assay was performed as previously described [20].

### Western blot

Briefly, whole-cell or tissue lysates were resolved by electrophoresis, and proteins were transferred to nitrocellulose membranes and blotted with antibodies against SPIN1 (1:1000, D152571, Sangon Biotech), ABCB4 (1:500, GTX47122, Genetex), CYP2C8 (1:200, sc-164136, Santa Cruz), UGT2B4 (1:2000, ab173580, Abcam), UGT2B17 (1:1000, abs110602, absin), or β-actin (1:1000, BA2305, Boster). The protein bands were detected using the chemiluminescent substrate with the AlphaView software (Version: 3.2.2.0) on a FluorChem Q machine (Cell Biosciences, Inc., Santa Clara, CA, USA).

### In vivo mouse xenograft model

Four-week-old female nude mice (*n* = 20) were used for xenograft studies. MCF-7/ADM cells (2 × 10^6^) were transplanted into the mammary fat pads of mice. We have previously shown that miRNA-489 could directly target and suppress SPIN1 expression in breast cancer [11]. Here we used miRNA-489 as a tool to downregulate SPIN1 expression in MCF-7/ADM cells. Lentiviruses miRNA-489 or miRNA control was stably transfected into cells. Chemosensitivity to Adriamycin was ascertained from mice injected with Adriamycin (5 mg/kg) through the tail vein weekly. Tumor growth was monitored once a week by Vernier caliper. At the 8th week, tumor masses were excised and further analyzed by immunohistochemistry, qRT-PCR and Western blot. Animal experiments were approved by the Laboratory Animal Center of Shandong University, and were conducted in accordance with the institutional guidelines.

### Statistical analysis

Statistical analysis was performed using GraphPad Prism 5 software. The correlation between miR-148/152 family, SPIN1, and *ABCB4/CYP2C8/UGT2B4/UGT2B17* expression was determined by Spearman’s correlation. Student’s *t* test was used to analyze the differences between two groups. The chi-square test or Fisher’s exact test was utilized to assess the relationship between SPIN1 expression and the clinical characteristics. *P* values < 0.05 were considered to be statistically significant.

## Results

### SPIN1 was elevated in drug-resistant breast cancer cells and tissues

**Fig. 1:**
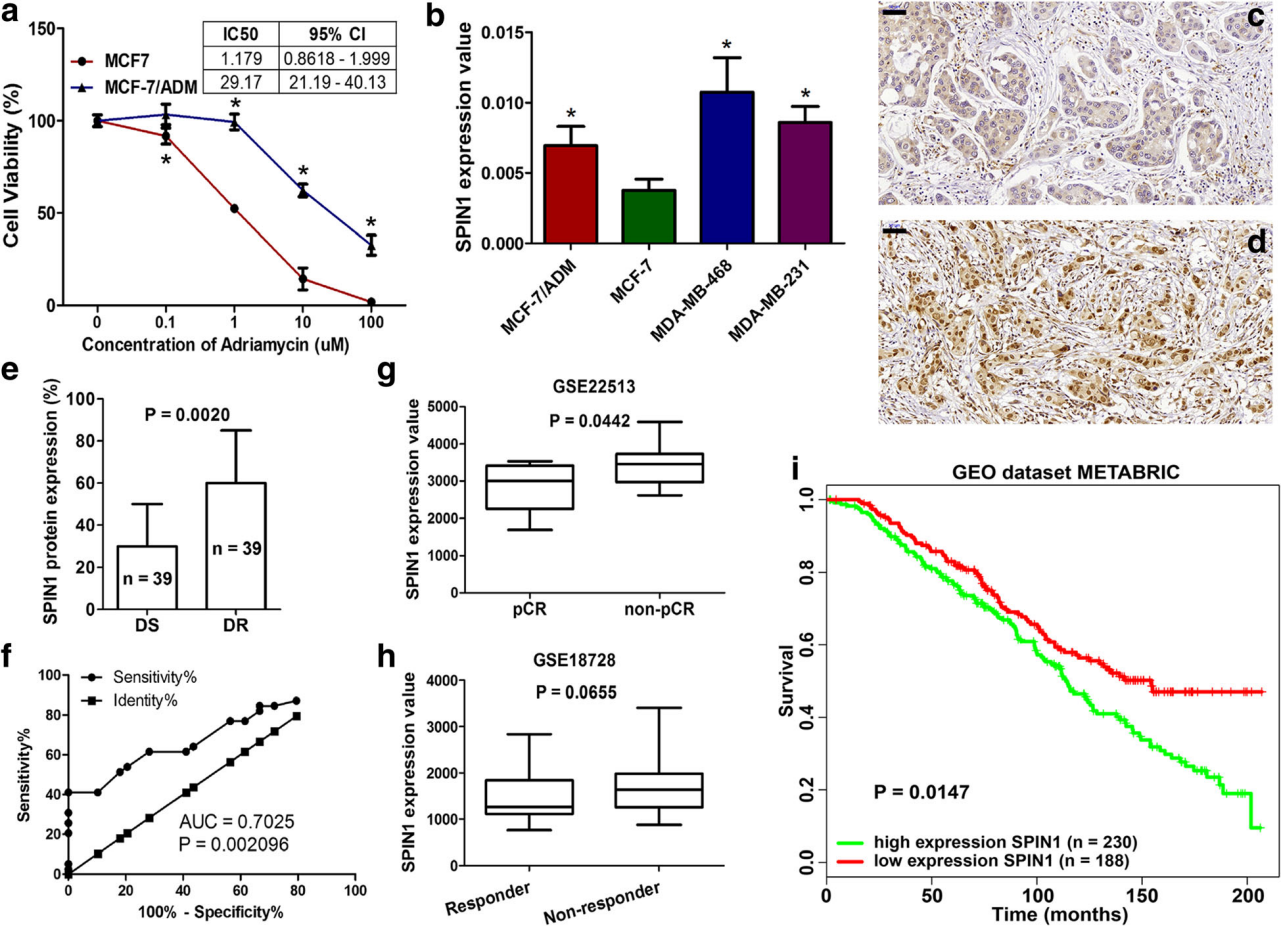
Expression of SPIN1 in breast cancer cells and tissues. **a** The Adriamycin resistant breast cancer MCF-7/ADM cells displayed greater resistance to Adriamycin than MCF-7 cells (IC50: 29.17 vs 1.179). **b** *SPIN1* was highly expressed in Adriamycin resistant MCF-7/ADM cells, and metastatic MDA-MB-231 and MDA-MB-468 cells, compared with MCF-7 cells (^*^P < 0.05). **c-e** Drug-resistant (**d**) tissues displayed higher SPIN1 protein levels than drug-sensitive (**c**) tissues (**e**, *P* = 0.0020, scale bar = 50 μm). **f** Receiver operating characteristic (ROC) curve analysis showed that SPIN1 could significantly distinguish drug-resistant tissues from drug-sensitive tissues (area under the curve (AUC) = 0.7025, *P* = 0.002096). **g** Data from GEO datasets GSE22513 showed that *SPIN1* was highly expressed in non-pCR patients compared to pCR (pathologic complete response) patients. **h** Data from GSE18728 showed that *SPIN1* expression was higher in non-responders than responders. **i** Analysis of the GEO dataset METABRIC showed that high expression of *SPIN1* predicted poorer survival of breast cancer patients

We have confirmed that the Adriamycin resistant breast cancer MCF-7/ADM cells displayed a more than 24-fold greater resistance to Adriamycin than the parental MCF-7 cells (Fig. 1a). *SPIN1* mRNA expression level was significantly upregulated in MCF-7/ADM cells compared with MCF-7 cells (Fig. 1b). Interestingly, SPIN1 was relatively highly expressed in triple-negative metastatic cell line MDA-MB-231 and MDA-MB-468, compared with the luminal cell line MCF-7 (Fig. 1b). This observation was consistent with the previous findings that high SPIN1 expression was associated with increased aggressiveness of breast cancer [11]. Additionally, we utilized GOBO [21] to determine the expression of *SPIN1* across a panel of commonly used breast cancer cell lines [22] and the analysis also revealed that *SPIN1* expression was significantly highly expressed in basal-like or triple-negative breast cancer cells (Additional file 1: Figure S1).

Next, we confirmed the correlation between SPIN1 expression and drug response in patients with breast cancer. Our results showed that drug-resistant breast cancer tissues (*n* = 39) exhibited higher SPIN1 protein levels compared with drug-sensitive tissues (*n* = 39) (Fig. 1c-f). Furthermore, the results showed positive relationship between SPIN1 expression and pN classification (Table 1, *P* = 0.035) and tumor stage (*P* = 0.020). Additionally, we determined the potential involvement of *SPIN1* in breast cancer chemoresistance based on publicly available data. We obtained the normalized data of the GEO datasets GSE22513 and GSE18728 from the GENT database [23]. It was revealed that *SPIN1* was upregulated in the pre-chemotherapy biopsies from patients who did not achieved a pathologic complete response (non-pCR), compared with those achieved a pathologic complete response (pCR) (GSE22513, Fig. 1g, *P* = 0.0442). Similarly, in the GEO dataset GSE18728, breast cancer patients who received neoadjuvant chemotherapy and classified as non-responders [24] displayed marginally higher *SPIN1* expression than those classified as responders (Fig. 1h, *P* = 0.0655). The prognostic role of *SPIN1* was determined by the PPISURV (http://www.chemoprofiling.org/cgi-bin/GEO/cancertarget/web_run_CT.V0.S1.pl) database [25, 26]. The results showed that patients with high expression of *SPIN1* (*n* = 230) had poorer survival than those with low expression of *SPIN1* (Fig. 1i, *n* = 188, *P* = 0.0147, GEO dataset METABRIC).

**Table 1:** Correlation between SPIN1 expression and clinicopathological features

Variables	SPIN1		*P* value
	Low	High	
Age (years)			
≤ 50	19	21	
> 50	24	14	0.180
Tumor size (cm)			
< 3	20	12	
≥ 3	23	23	0.356
Lymph node metastases			
Negative	12	4	
Positive	31	31	0.094
Chemosensitivity			
Drug-sensitive	28	11	
Drug-resistant	15	24	0.006
ER			
Negative	18	9	
Positive	25	26	0.158
PR			
Negative	22	13	
Positive	21	22	0.257
HER2			
Negative	24	20	
Positive	13	9	0.796
Missing	12		
Ki-67			
Negative (< 14%)	13	9	
Positive (≥14%)	30	26	0.801
pT			
0	2	0	
I	13	11	
II	24	16	
III	3	8	
IV	1	0	0.179
pN			
0	12	5	
I	13	4	
II	11	14	
III	7	12	0.035
Distant metastases			
M0	40	34	
M1	3	1	0.623
pTNM stage			
I + II	23	9	
III + IV	20	26	0.020

### SPIN1 enhanced breast cancer resistance to Adriamycin in vitro and in vivo

**Fig. 2:**
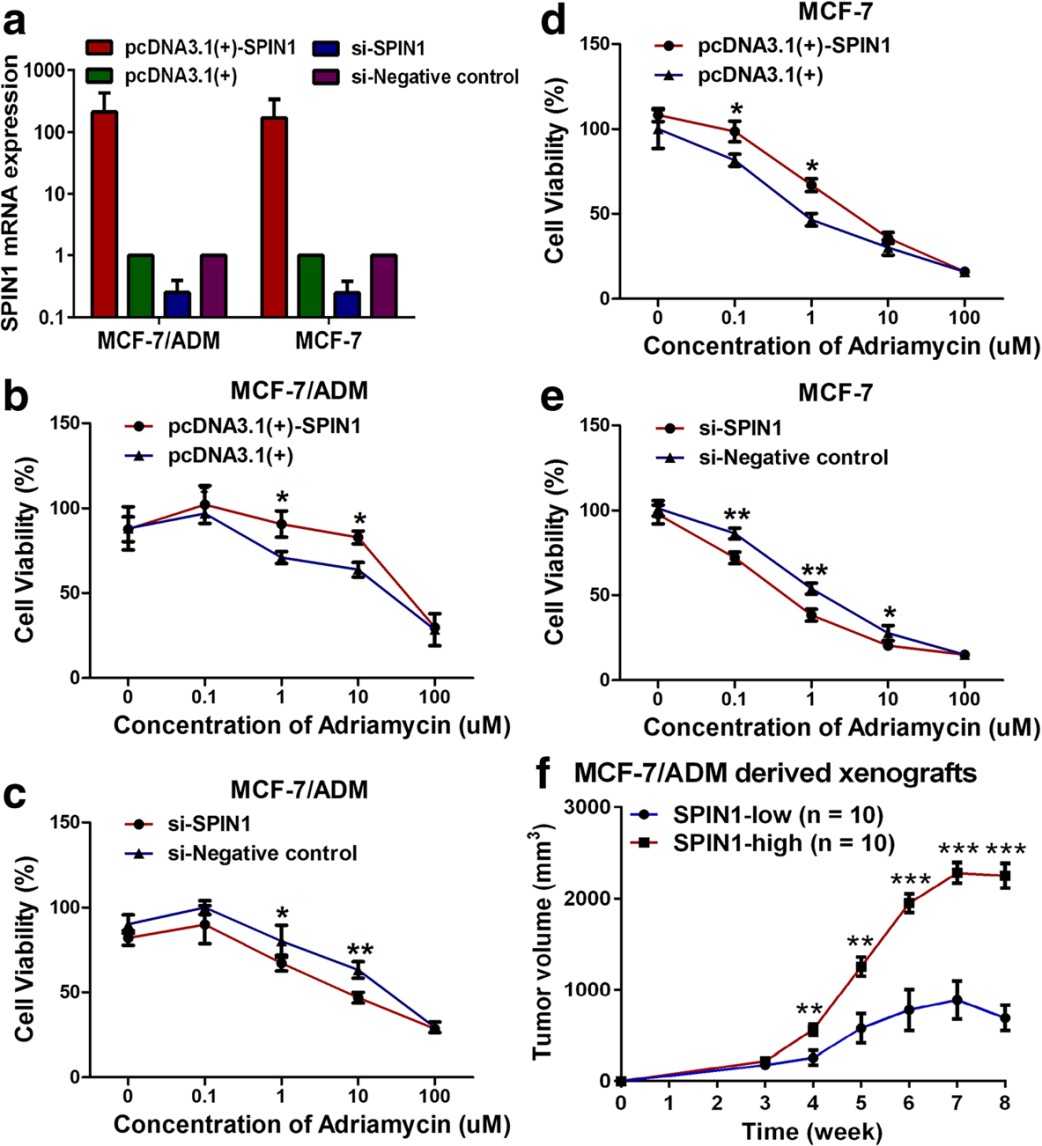
SPIN1 increased breast cancer resistance to Adriamycin in vitro and in vivo. **a** *SPIN1* overexpression plasmid transfection increased, whereas *SPIN1* siRNA transfection decreased, *SPIN1* mRNA expression in breast cancer cells. **b**, **d** Overexpression of *SPIN1* enhanced chemoresistance in MCF-7/ADM and MCF-7 cells. **c, e** Downregulation of *SPIN1* increased chemosensitivity in MCF-7/ADM and MCF-7 cells. **f** Tumors originating from MCF-7/ADM cells expressing miRNA against *SPIN1* (SPIN1-low) were significantly smaller in size than those originating from control (SPIN1-high) cells (^**^P < 0.01, ^***^P < 0.001)

To investigate the role of SPIN1 in breast cancer Adriamycin resistance, the effect of SPIN1 overexpression or inhibition on cell viability was measured by drug-sensitivity assay. As shown in Fig. 2a-c, overexpression of *SPIN1* conferred greater Adriamycin resistance to MCF-7/ADM cells, with more than twofold increase in the IC50 values (43.17 μM vs 18.41 μM). However, *SPIN1* knockdown in MCF-7/ADM cells significantly decreased the IC50 value by more than twofold (8.004 μM vs 22.19 μM). These findings were further confirmed in MCF-7 cells (Fig. 2d-e).

To investigate whether SPIN1 enhanced resistance of breast tumor to Adriamycin in vivo, MCF-7/ADM cells stably expressing miRNA (or control) against *SPIN1* were injected into mammary fat pad of female mice. Tumors were allowed to grow for 3 weeks. Subsequently, the nude mice were administered with Adriamycin, and the volumes of tumors of mice were measured. After administration with Adriamycin, the tumors originating from cells expressing miRNA against *SPIN1* (SPIN1-low) [11] were significantly smaller in size than those tumors originating from control (SPIN1-high) cells (Fig. 2f). These results indicated that SPIN1 increased breast cancer cells resistance to Adriamycin in vivo.

### Drug metabolizing enzymes CYP2C8, UGT2B4 and UGT2B17, and drug transporter ABCB4 were activated by SPIN1 in breast cancer

**Fig. 3:**
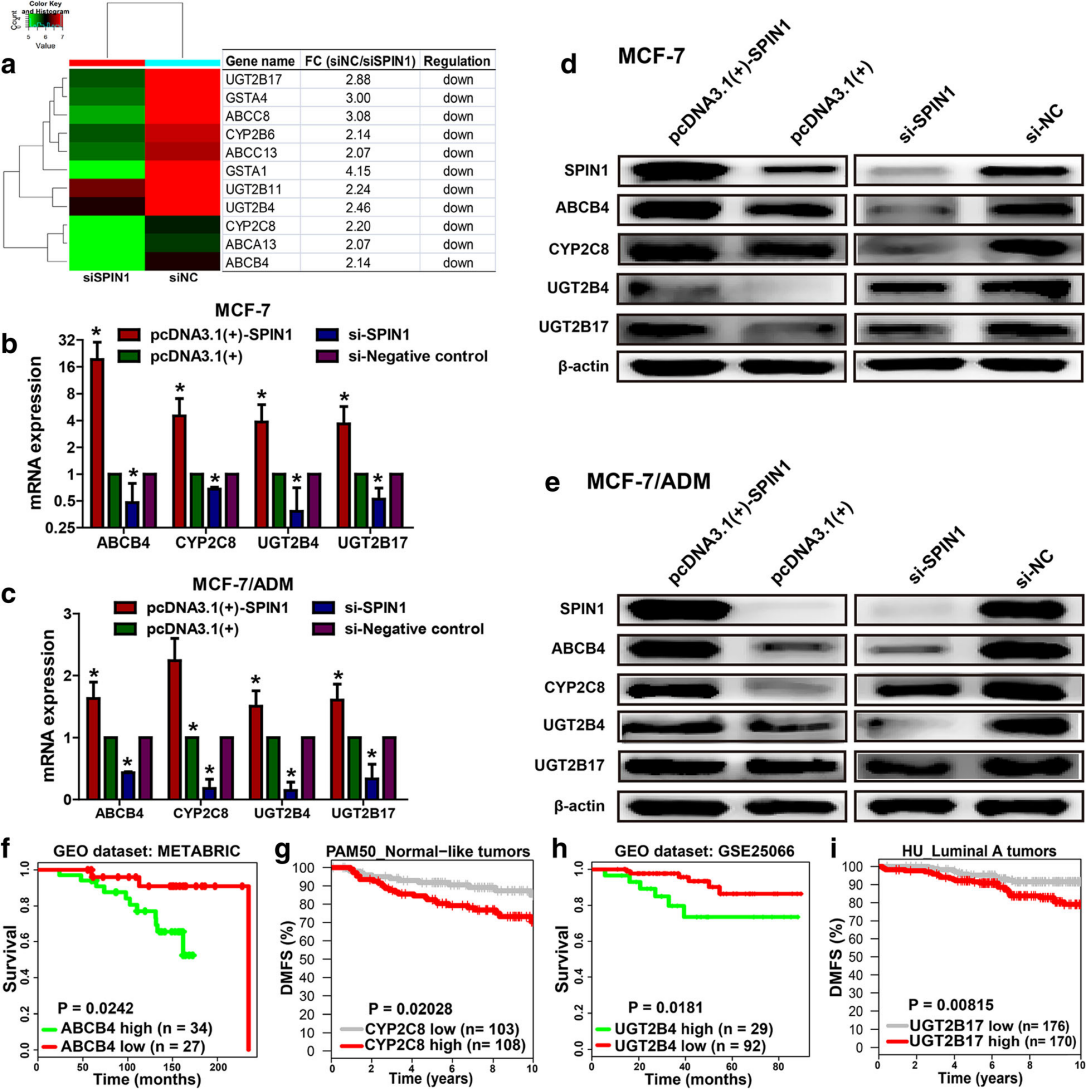
The expression of ABCB4, CYP2C8, UGT2B4 and UGT2B17 was upregulated by SPIN1 in breast cancer cells. **a** Candidate *SPIN1* targets from microarray were presented as a heat map. **b-c** The mRNA expression levels of *ABCB4*, *CYP2C8*, *UGT2B4*, and *UGT2B17* were decreased after *SPIN1* siRNA transfection. Accordingly, overexpression of *SPIN1* led to upregulation of *ABCB4*, *CYP2C8*, *UGT2B4*, and *UGT2B17* mRNA expression levels. **d-e** Western blot analysis confirmed that overexpression of SPIN1 upregulated ABCB4, CYP2C8, UGT2B4, and UGT2B17 protein levels, however, SPIN1 downregulation decreased the expression levels of these proteins. **f-i** Data from PPISURV (*ABCB4* and *UGT2B4*) and GOBO (*CYP2C8* and *UGT2B17*) revealed that patients with high expression of *ABCB4*, *CYP2C8*, *UGT2B4* or *UGT2B17* had poorer survival than those with low expression

In order to identify the downstream molecules regulated by SPIN1, MCF-7/ADM cells transfected with *SPIN1* siRNA (or negative control) were subjected to gene expression microarray**.** Here we focused on differentially-expressed genes (fold change > 2, *P* < 0.05) that would affect drug metabolism or transport (Fig. 3a). Thus we selected members of ATP-binding cassette (ABC) transporters (*ABCA13, ABCB4, ABCC13* and *ABCC8*) and genes that enriched in the “drug metabolism - cytochrome P450” pathway (*CYP2B6*, *CYP2C8*, *GSTA1*, *GSTA4*, *UGT2B11*, *UGT2B17* and *UGT2B4*, *P* = 0.01) for qRT-PCR validation. Results showed that the mRNA expression levels of *ABCB4*, *CYP2C8*, *UGT2B4*, and *UGT2B17* were significantly decreased after *SPIN1* siRNA transfection (Fig. 3b-c). Reciprocally, overexpression of SPIN1 enhanced the *ABCB4*, *CYP2C8*, *UGT2B4* and *UGT2B17* mRNA expression when compared with the negative control (Fig. 3b-c). Western blot was further used to detect the protein levels of these drug metabolizing enzymes and transporters. We found that after transfection with *SPIN1* siRNA, levels of ABCB4, CYP2C8, UGT2B4, and UGT2B17 were dramatically reduced as compared to the negative controls (Fig. 3d-e). Consistently, the protein levels in the SPIN1-overexpressing cells showed significant increases as compared with that of the control cells (Fig. 3d-e). Altogether these data indicate that the expression of the drug metabolizing enzymes CYP2C8, UGT2B4 and UGT2B17, and transporter ABCB4 was positively regulated by SPIN1 in breast cancer.

Data from PPISURV [25, 26] and GOBO [21] revealed that breast cancer patients with high expression of *ABCB4* (*P* = 0.0242), *CYP2C8* (*P* = 0.02028), *UGT2B4* (*P* = 0.0181) or *UGT2B17* (*P* = 0.00815) showed poorer survival than those with low expression (Fig. 3f-i).

### *SPIN1* was directly targeted and suppressed by the miR-148/152 family

MicroRNAs (miRNAs) are critical gene regulators and chemotherapy modifiers in different tumor types [27]. We suspected that *SPIN1* may be potentially regulated by miRNAs and we utilized the TargetScan algorithm to screen chemotherapy-associated miRNAs that target the 3’-UTR of *SPIN1* mRNA. Of the candidates, the miR-148/152 family (miR-148a-3p, miR-148b-3p and miR-152-3p), was of particular interest in light of its reported roles in regulating drug sensitivity of cancer cells [28–30]. Moreover, a microarray analysis by Kovalchuk et al. found that two members (miR-148a and miR-152) of the family displayed more than 370-fold decreases in MCF-7/ADM cells versus MCF-7 cells [31], further indicating their possible involvement in breast cancer chemoresistance.

**Fig. 4:**
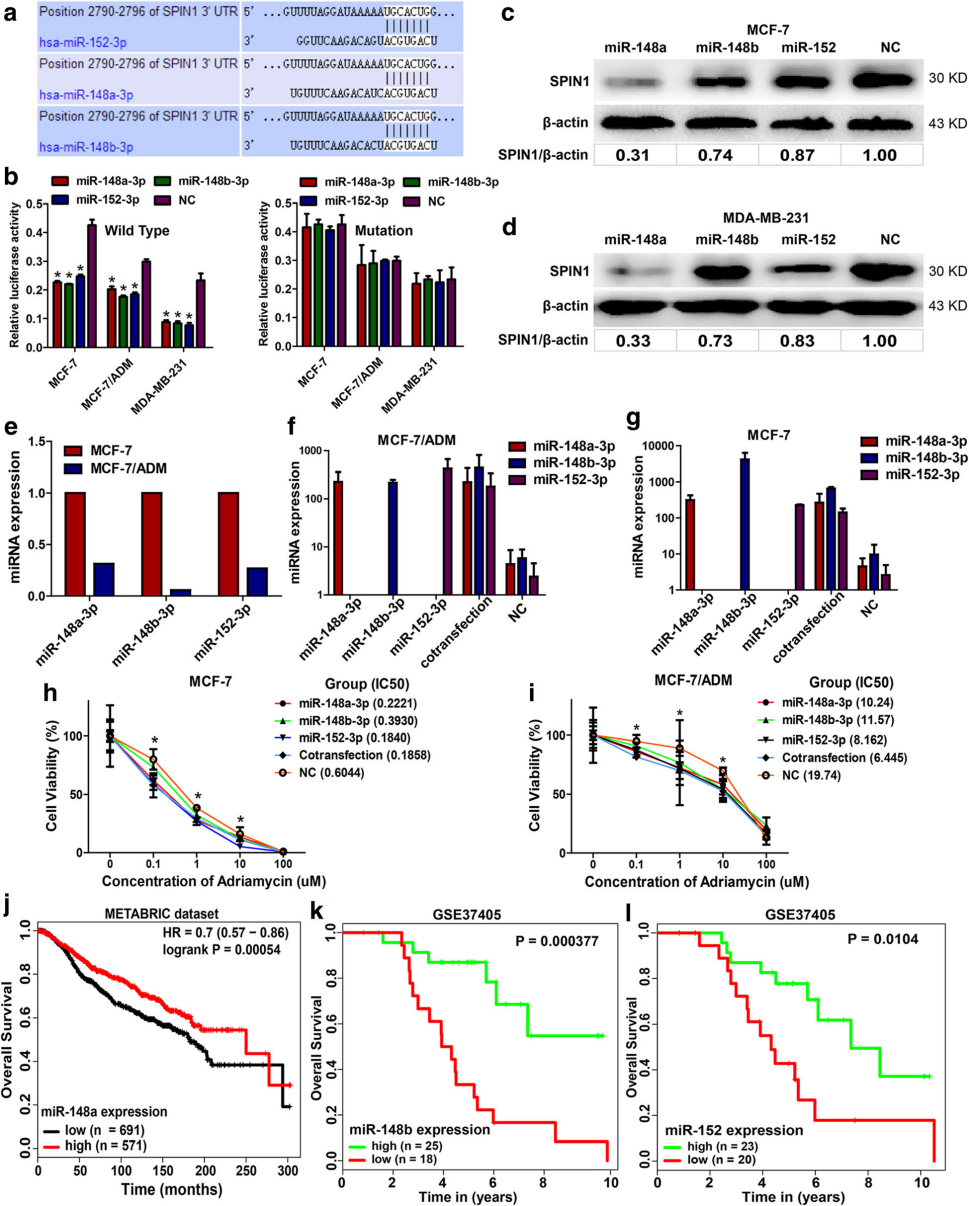
miR-148a-3p, miR-148b-3p and miR-152-3p directly targeted *SPIN1* and increased chemosensitivity in breast cancer cells**. a** The 3’-UTR element of *SPIN1* mRNA is partially complementary to miR-148a-3p, miR-148b-3p and miR-152-3p. **b** The relative luciferase activity was significantly reduced in the miR-148a/148b/152-3p overexpressing cells (^*^P < 0.05) and these effects could be abolished by mutation of *SPIN1* 3’-UTR. **c-d** The SPIN1 protein expression was clearly reduced after the transfection of miR-148a-3p, miR-148b-3p or miR-152-3p. **e** The expression levels of miR-148a-3p, miR-148b-3p and miR-152-3p were lower in MCF-7/ADM cells than that in MCF-7 cells. **f-g** Transfection of miR-148a-3p, miR-148b-3p, miR-152-3p, or cotransfection of these three miRNAs significantly increased the miRNAs levels. **h-i** miR-148a-3p, miR-148b-3p and miR-152-3p decreased Adriamycin resistance in MCF-7/ADM and MCF-7 cells. In MCF-7 cells, miR-148a-3p or miR-152-3p (Adriamycin = 0.1 μM and 1 μM), and miR-152-3p (Adriamycin = 10 μM) could significantly reduce cell viability, compared with the negative control (NC) group (*P < 0.05, t-test). In MCF-7/ADM cells, miR-148a-3p or miR-152-3p (Adriamycin = 0.1 μM), and miR-148a-3p, miR-148b-3p or miR-152-3p (Adriamycin = 1 μM and 10 μM) could significantly decrease cell viability compared with NC (*P < 0.05, t-test). **j-l** Data from MIRUMIR and miRpower showed that breast cancer patients with low expression of miR-148a, miR-148b or miR-152 had shorter survival time than those with high expression

By luciferase assay, we observed a significant suppression of luciferase activity in cells transfected with miR-148a-3p, miR-148b-3p or miR-152-3p (Fig. 4a-b). On the contrary, when the binding sites of these three miRNAs in the *SPIN1* pmirGLO-3’UTR were mutated, its responsiveness to miR-148a-3p, miR-148b-3p or miR-152-3p regulation was abrogated (Fig. 4b). We further investigated the miR-148/152 family-mediated SPIN1 suppression and found that expression of the SPIN1 protein was significantly downregulated in miR-148/152 overexpressing MCF-7 and MDA-MB-231 cells (Fig. 4c-d). Interestingly, downregulation of SPIN1 protein is significative after overexpression of miR-148a-3p in particular (Fig. 4c-d).

### The miR-148/152 family decreased Adriamycin resistance in breast cancer cells and was associated with patients’ survival

Our results showed the chemoresistant MCF-7/ADM cells exhibited lower miR-148a-3p, miR-148b-3p and miR-152-3p expression levels compared with chemosensitive MCF-7 cells (Fig. 4e). We further characterized the roles of miR-148a-3p, miR-148b-3p and miR-152-3p in regulating Adriamycin resistance. Transfection of miR-148a-3p, miR-148b-3p or miR-152-3p resulted in a significant decrease in survival of MCF-7/ADM and MCF-7 cells in Adriamycin-added medium (Fig. 4f-i).

Furthermore, we determined the prognostic value of the miR-148/152 family using publicly available data. As shown in MIRUMIR [26, 32], low expression of miR-148b (*P* = 0.000377) or miR-152 (*P* = 0.0104) predicted poor survival of breast cancer patients (GEO database: GSE37405, *n* = 43). Results from the web-tool miRpower [33] showed that breast cancer patients with low expression of miR-148a had shorter survival time (METABRIC dataset, *n* = 1262, *P* = 0.00054).

### Validation of the miR-148/152–SPIN1–ABCB4/CYP2C8/UGT2B4/UGT2B17 signaling axis in xenograft tumors

**Fig. 5:**
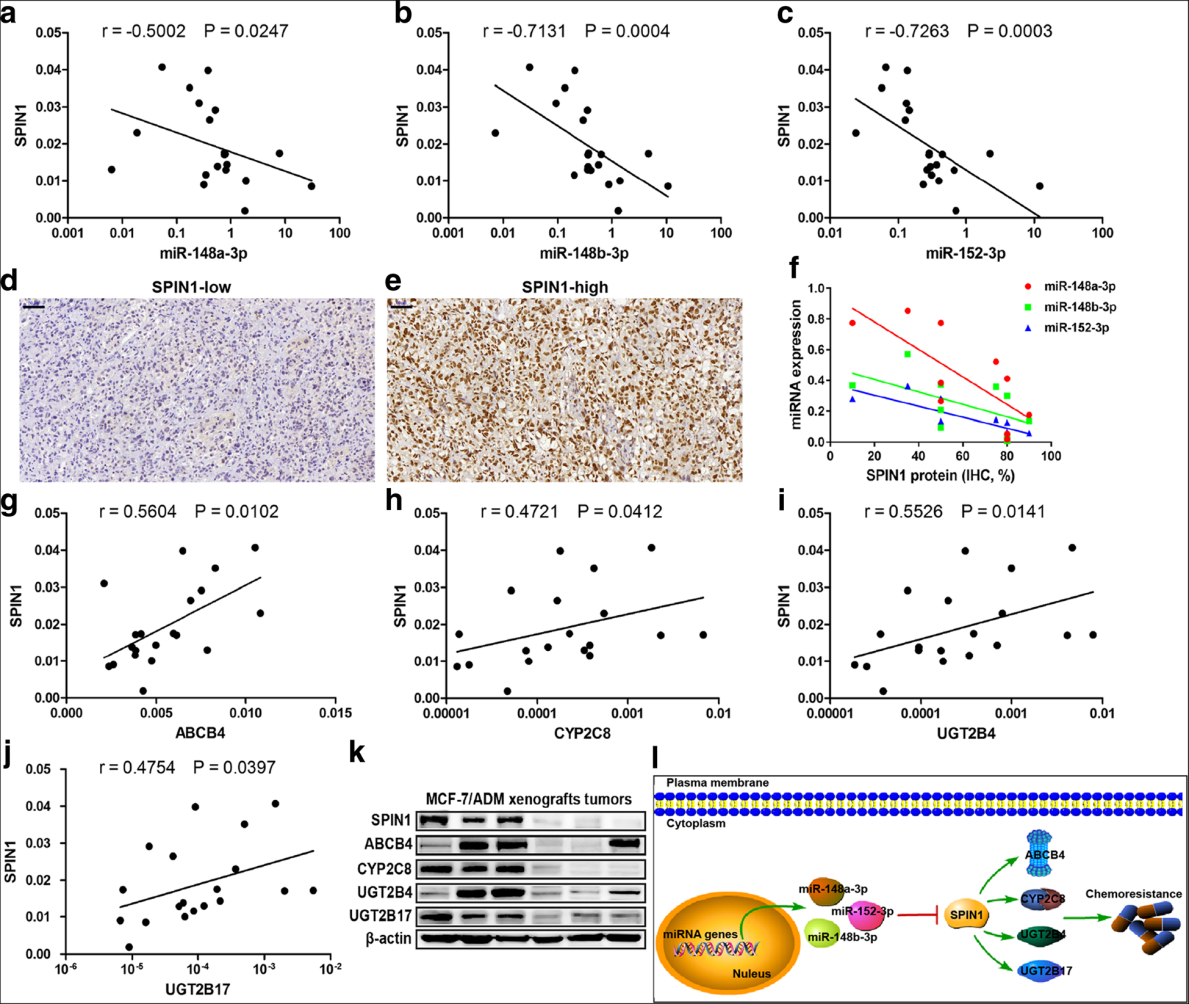
Validation of the miR-148/152–SPIN1–ABCB4/CYP2C8/UGT2B4/UGT2B17 signaling in xenograft tumors. **a-c** Expression levels of miR-148a-3p, miR-148b-3p or miR-152-3p were inversely related with *SPIN1* mRNA expression in MCF-7/ADM xenograft tumors (*n* = 20). **d-e** SPIN1 protein expression in ten xenograft tumors was detected by immunohistochemistry. Representative images were shown. **f** Expression of miR-148a-3p (*r* = − 0.7478, *P* = 0.0162), miR-148b-3p (*r* = − 0.6524, *P* = 0.0473) or miR-152-3p (*r* = − 0.8512, *P* = 0.0032) were inversely related with SPIN1 protein expression. **g-j** A positive relationship between mRNA expression of *SPIN1* and *ABCB4*, *CYP2C8*, *UGT2B4* and *UGT2B17* was observed (*n* = 20). **k** Three tumors with high expression of SPIN1 and the other three with low SPIN1 expression were selected for Western blot analysis. Tumors with high SPIN1 protein levels tend to show high expression of ABCB4, CYP2C8, UGT2B4 and UGT2B17. **l** A schematic model of miR-148/152–SPIN1–ABCB4/CYP2C8/UGT2B4/UGT2B17 regulation of breast cancer chemoresistance. SPIN1, which is direct target of miR-148a-5p/148b-5p/152-5p, promotes chemoresistance via upregulating ABCB4, CYP2C8, UGT2B4 and UGT2B17

In order to validate the miR-148/152**–**SPIN1**–**downstream effectors axis in vivo, we established an MCF-7/ADM derived xenograft tumor model, by suppressing SPIN1 expression [11]. Analysis on the xenograft tumors showed that expression of miR-148a-3p, miR-148b-3p or miR-152-3p was positively intercorrelated (Additional file 1: Figure S2). Moreover, expression levels of the miR-148/152 family were inversely related with SPIN1 mRNA (Fig. 5a-c) and protein (Fig. 5d-f) expression.

As for the drug metabolizing enzymes and transporter, we observed a positive relationship between mRNA expression of *SPIN1* and *ABCB4*, *CYP2C8*, *UGT2B4* and *UGT2B17* in the xenograft tumors (Fig. 5g-j). As expected, tumors with high SPIN1 protein levels displayed relatively higher protein expression of ABCB4, CYP2C8, UGT2B4 and UGT2B17, compared to tumors with low SPIN1 protein levels (Fig. 5k).

## Discussion

Here, we show that SPIN1 is upregulated in drug-resistant breast cancer cells and tissues. *SPIN1* is identified as a novel target of the miR-148/152 family and enhances Adriamycin resistance by regulating drug metabolizing enzymes and transporter CYP2C8, UGT2B4, UGT2B17 and ABCB4 in breast cancer (Fig. 5i). In addition, high expression of *SPIN1* or low expression of the miR-148/152 family predicts poorer survival in patients with breast cancer.

In this study, by ectopic expression and loss-of-function experiments of SPIN1, we indicated that SPIN1 enhanced Adriamycin resistance of breast cancer cells. We understand that in vivo experiments with a specific depletion of SPIN1 may better reveal the involvement of SPIN1 in chemoresistance, which needs more attention in our future study. Here SPIN1 was found to lead to marked upregulation of drug metabolizing enzymes and transporter. Recently, Franz et al. have found that SPIN1, in cooperation with the transcription factor MAZ, directly enhances expression of GDNF to activate the RET signaling to increase proliferation and decrease apoptosis of liposarcoma cells [10]. These findings, together with our results, highlighted the critical roles of SPIN1 in regulating genes expression and prompted us to further investigate the underlying mechanisms of SPIN1-mediated activation of the drug metabolizing enzymes and transporter.

The drug metabolizing enzymes and transporter that identified as SPIN1 downstream effectors in this study have been shown to be highly expressed in drug-resistant tumors and associated with chemoresistance. The ATP-binding cassette (ABC) transporter gene *ABCB4* is found to be amplified in Adriamycin-resistant breast cancer cells relative to drug-sensitive cells [34]. Sprouse et al. suggest that upregulation of CYP2C8 may account for paclitaxel resistance in the drug-resistant MDA-MB-231 breast cancer cells [35]. The UDP-glucuronosyltransferases UGT2B4 and UGT2B17 have been linked to breast cancer risk [36, 37]. However the upstream regulators of these drug metabolizing enzymes and transporter remain largely unknown. Here we showed that these drug metabolizing enzymes and transporter were controlled by SPIN1 and involved in breast cancer chemoresistance, which may be potential targets to reverse drug resistance in breast cancer.

We have previously found that miR-489 could directly target *SPIN1* in breast cancer [11]. And here we further showed that *SPIN1* was also targeted by three members (miR-148a/148b/152-3p) of the miR-148/152 family [38]. All these three miRNAs have been reported to be downregulated in breast cancer tissues and cell lines [39–41]. In addition, they are all crucial modulators for many biological processes in breast cancer. MiR-148a inhibits breast cancer migration, invasion and angiogenesis by suppressing *WNT-1* [39], *MMP-13* [42], *ERBB3* [43]. MiR-148b is identified as a relapse-associated miRNA and suppresses breast cancer progression by targeting a series of cancer-related oncogenes [40]. MiR-152 inhibits tumor angiogenesis via targeting *IGF-IR* and *IRS1* in breast cancer [41]. However, the function and mechanism of the miR-148/152 family in breast cancer chemoresistance have not yet been reported. Here we found that miR-148-3p, miR-148b-3p and miR-152-3p were downregulated in Adriamycin-resistant MCF-7/ADM cells compared with the parental MCF-7 cells. The three miRNAs suppressed Adriamycin resistance of breast cancer cells by directly targeting *SPIN1*. Moreover, analysis of publicly available data revealed that the miR-148/152 family was associated with patients’ survival in breast cancer. Our study demonstrated a newly-identified involvement of the miR-148/152 family in breast cancer Adriamycin resistance and further study is underway to confirm this in clinical samples.

In conclusion, we have presented evidence that SPIN1, a novel target of the miR-148/152 family, is upregulated in drug-resistant breast cancer cells and tissues and confers Adriamycin resistance by upregulating drug metabolizing enzymes and transporter in breast cancer. Our study may provide useful information for the development of alternative approaches to drug-resistant breast cancer.

## Additional file

Additional file 1: **Figure S1 and Figure S2.** SPIN1 expression in breast cancer cells and miR-148a-3p/148b-3p/152-3p expression in xenograft tumors. (DOC 261 kb)

## Abbreviations

FBS: Fetal bovine serum
GENT database: Gene Expression database of Normal and Tumor tissues database
GEO: Gene Expression Omnibus
GSTA4: Glutathione S-transferase alpha 4
H3K4me3: Histone H3 trimethylated at lysine 4
miRNAs: MicroRNAs
PBS: Phosphate buffer saline
pCR: Pathologic complete response
PI3K/Akt: Phosphatidylinositol 3 kinase/protein kinase B
qRT-PCR: Quantitative real-time PCR
SP: Streptavidin-peroxidase-biotin
SPIN1: Spindlin1
UGT2B4: UDP glucuronosyltransferase family 2 member B4

## References

[1] Gao P, Zhou GY, Guo LL, Zhang QH, Zhen JH, Fang AJ, Lin XY (2007). Reversal of drug resistance in breast carcinoma cells by anti-mdr1 ribozyme regulated by the tumor-specific MUC-1 promoter. Cancer Lett.

[2] Starlard-Davenport A, Lyn-Cook B, Beland FA, Pogribny IP (2010). The role of UDP-glucuronosyltransferases and drug transporters in breast cancer drug resistance. Exp Oncol.

[3] Cascorbi I (2006). Role of pharmacogenetics of ATP-binding cassette transporters in the pharmacokinetics of drugs. Pharmacol Ther.

[4] Wang C, Liu KX (2014). The drug-drug interaction mediated by efflux transporters and CYP450 enzymes. Yao Xue Xue Bao.

[5] Decleves X, Jacob A, Yousif S, Shawahna R, Potin S, Scherrmann JM (2011). Interplay of drug metabolizing CYP450 enzymes and ABC transporters in the blood-brain barrier. Curr Drug Metab.

[6] Vadlapatla RK, Vadlapudi AD, Pal D, Mitra AK (2013). Mechanisms of drug resistance in cancer chemotherapy: coordinated role and regulation of efflux transporters and metabolizing enzymes. Curr Pharm Des.

[7] Oh B, Hwang SY, Solter D, Knowles BB (1997). Spindlin, a major maternal transcript expressed in the mouse during the transition from oocyte to embryo. Development.

[8] Yue W, Sun LY, Li CH, Zhang LX, Pei XT (2004). Screening and identification of ovarian carcinomas related genes. Ai Zheng.

[9] Wang JX, Zeng Q, Chen L (2012). SPINDLIN1 promotes cancer cell proliferation through activation of WNT/TCF-4 signaling. Mol Cancer Res.

[10] Franz H, Greschik H, Willmann D (2015). The histone code reader SPIN1 controls RET signaling in liposarcoma. Oncotarget.

[11] Chen X, Wang YW, Xing AY (2016). Suppression of SPIN1-mediated PI3K-Akt pathway by miR-489 increases chemosensitivity in breast cancer. J Pathol.

[12] Yang N, Wang W, Wang Y (2012). Distinct mode of methylated lysine-4 of histone H3 recognition by tandem tudor-like domains of Spindlin1. Proc Natl Acad Sci U S A.

[13] Wang W, Chen Z, Mao Z (2011). Nucleolar protein Spindlin1 recognizes H3K4 methylation and stimulates the expression of rRNA genes. EMBO Rep.

[14] Voigt P, Tee WW, Reinberg D (2013). A double take on bivalent promoters. Genes Dev.

[15] Lim LP, Lau NC, Garrett-Engele P (2005). Microarray analysis shows that some microRNAs downregulate large numbers of target mRNAs. Nature.

[16] Wu S, Huang S, Ding J (2010). Multiple microRNAs modulate p21Cip1/Waf1 expression by directly targeting its 3′ untranslated region. Oncogene.

[17] Ogston KN, Miller ID, Payne S (2003). A new histological grading system to assess response of breast cancers to primary chemotherapy: prognostic significance and survival. Breast.

[18] Kim YJ, Kim SH, Lee AW, Jin MS, Kang BJ, Song BJ (2016). Histogram analysis of apparent diffusion coefficients after neoadjuvant chemotherapy in breast cancer. Jpn J Radiol.

[19] Kim T, Kang DK, An YS (2014). Utility of MRI and PET/CT after neoadjuvant chemotherapy in breast cancer patients: correlation with pathological response grading system based on tumor cellularity. Acta Radiol.

[20] Wang YW, Chen X, Gao JW, Zhang H, Ma RR, Gao ZH, Gao P (2015). High expression of cAMP-responsive element-binding protein 1 (CREB1) is associated with metastasis, tumor stage and poor outcome in gastric cancer. Oncotarget.

[21] Ringner M, Fredlund E, Hakkinen J, Borg A, Staaf J (2011). GOBO: gene expression-based outcome for breast cancer online. PLoS One.

[22] Neve RM, Chin K, Fridlyand J (2006). A collection of breast cancer cell lines for the study of functionally distinct cancer subtypes. Cancer Cell.

[23] Shin G, Kang TW, Yang S, Baek SJ, Jeong YS, Kim SY (2011). GENT: gene expression database of normal and tumor tissues. Cancer Inform.

[24] Korde LA, Lusa L, McShane L (2010). Gene expression pathway analysis to predict response to neoadjuvant docetaxel and capecitabine for breast cancer. Breast Cancer Res Treat.

[25] Antonov AV, Krestyaninova M, Knight RA, Rodchenkov I, Melino G, Barlev NA (2014). PPISURV: a novel bioinformatics tool for uncovering the hidden role of specific genes in cancer survival outcome. Oncogene.

[26] Antonov AV (2011). BioProfiling.de: analytical web portal for high-throughput cell biology. Nucleic Acids Res.

[27] Hummel R, Hussey DJ, Haier J (2010). MicroRNAs: predictors and modifiers of chemo- and radiotherapy in different tumour types. Eur J Cancer.

[28] Hummel R, Watson DI, Smith C, Kist J, Michael MZ, Haier J, Hussey DJ (2011). Mir-148a improves response to chemotherapy in sensitive and resistant oesophageal adenocarcinoma and squamous cell carcinoma cells. J Gastrointest Surg.

[29] Sui C, Meng F, Li Y, Jiang Y (2015). miR-148b reverses cisplatin-resistance in non-small cell cancer cells via negatively regulating DNA (cytosine-5)-methyltransferase 1(DNMT1) expression. J Transl Med.

[30] Xiang Y, Ma N, Wang D (2014). MiR-152 and miR-185 co-contribute to ovarian cancer cells cisplatin sensitivity by targeting DNMT1 directly: a novel epigenetic therapy independent of decitabine. Oncogene.

[31] Kovalchuk O, Filkowski J, Meservy J, Ilnytskyy Y, Tryndyak VP, Chekhun VF, Pogribny IP (2008). Involvement of microRNA-451 in resistance of the MCF-7 breast cancer cells to chemotherapeutic drug doxorubicin. Mol Cancer Ther.

[32] Antonov AV, Knight RA, Melino G, Barlev NA, Tsvetkov PO (2013). MIRUMIR: an online tool to test microRNAs as biomarkers to predict survival in cancer using multiple clinical data sets. Cell Death Differ.

[33] Lanczky A, Nagy A, Bottai G, Munkacsy G, Szabo A, Santarpia L, Gyorffy B (2016). miRpower: a web-tool to validate survival-associated miRNAs utilizing expression data from 2178 breast cancer patients. Breast Cancer Res Treat.

[34] Turton NJ, Judah DJ, Riley J (2001). Gene expression and amplification in breast carcinoma cells with intrinsic and acquired doxorubicin resistance. Oncogene.

[35] Sprouse AA, Herbert BS (2014). Resveratrol augments paclitaxel treatment in MDA-MB-231 and paclitaxel-resistant MDA-MB-231 breast cancer cells. Anticancer Res.

[36] Sun C, Huo D, Southard C (2011). A signature of balancing selection in the region upstream to the human UGT2B4 gene and implications for breast cancer risk. Hum Genet.

[37] Eskandari-Nasab E, Hashemi M, Rezaei H (2012). Evaluation of UDP-glucuronosyltransferase 2B17 (UGT2B17) and dihydrofolate reductase (DHFR) genes deletion and the expression level of NGX6 mRNA in breast cancer. Mol Biol Rep.

[38] Chen Y, Song YX, Wang ZN (2013). The microRNA-148/152 family: multi-faceted players. Mol Cancer.

[39] Jiang Q, He M, Ma MT (2016). MicroRNA-148a inhibits breast cancer migration and invasion by directly targeting WNT-1. Oncol Rep.

[40] Cimino D, De Pitta C, Orso F (2013). miR148b is a major coordinator of breast cancer progression in a relapse-associated microRNA signature by targeting ITGA5, ROCK1, PIK3CA, NRAS, and CSF1. FASEB J.

[41] Xu Q, Jiang Y, Yin Y (2013). A regulatory circuit of miR-148a/152 and DNMT1 in modulating cell transformation and tumor angiogenesis through IGF-IR and IRS1. J Mol Cell Biol.

[42] Xue J, Chen Z, Gu X, Zhang Y, Zhang W (2016). MicroRNA-148a inhibits migration of breast cancer cells by targeting MMP-13. Tumour Biol.

[43] Yu J, Li Q, Xu Q, Liu L, Jiang B (2011). MiR-148a inhibits angiogenesis by targeting ERBB3. J Biomed Res.

