# Supplementary material for: SPIN1, negatively regulated by miR-148/152, enhances Adriamycin resistance via upregulating drug metabolizing enzymes and transporter in breast cancer
Source: J Exp Clin Cancer Res. 2018 May 9;37:100. doi: 10.1186/s13046-018-0748-9 (PMC5944004; doi:10.1186/s13046-018-0748-9)
Supplement: Supplementary file 1 — Figure S1 and Figure S2. SPIN1 expression in breast cancer cells and miR-148a-3p/148b-3p/152-3p expression in xenograft tumors. (DOC 261 kb) [file 13046_2018_748_MOESM1_ESM.doc]

**Supplementary Material**

**
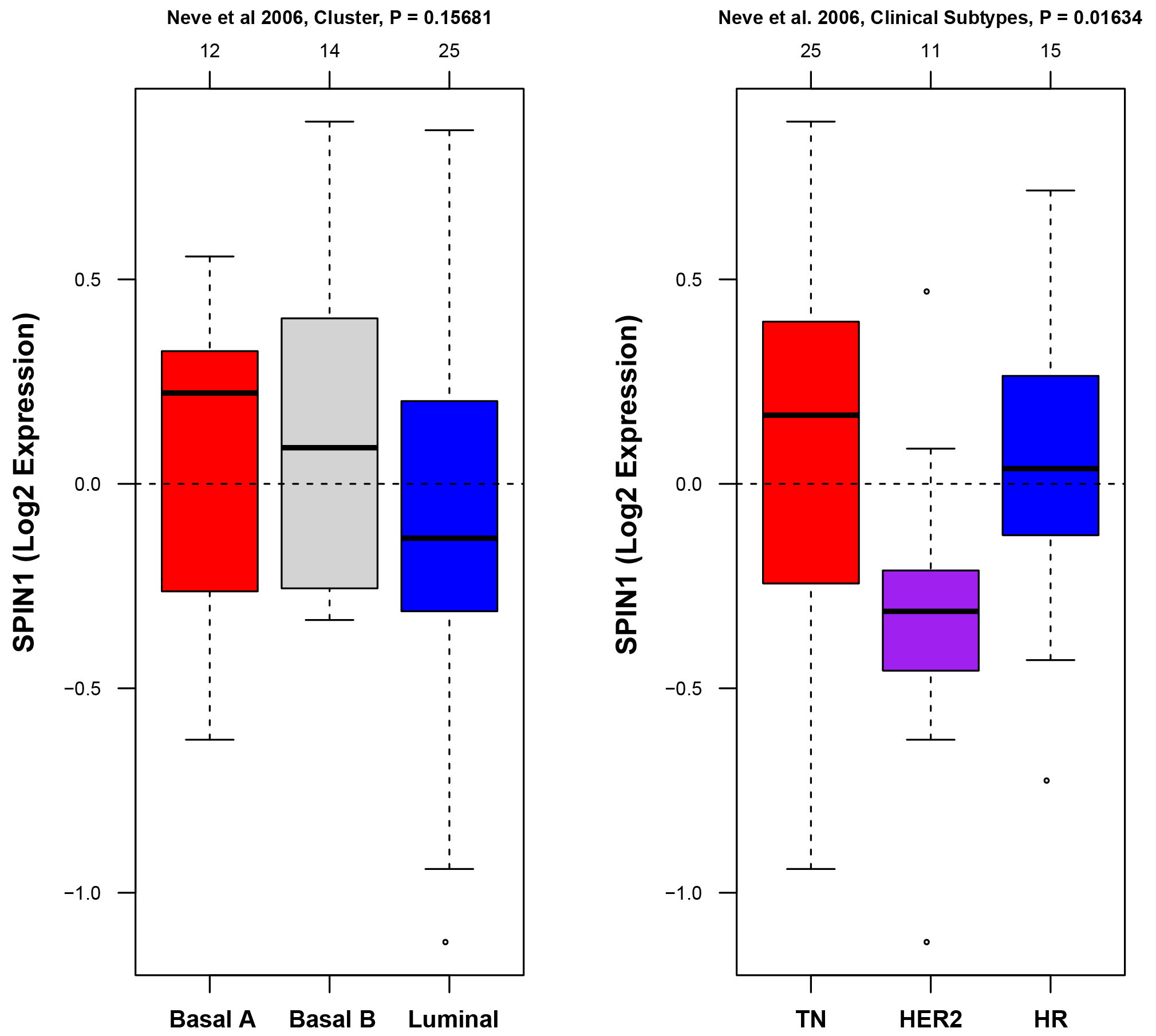
**

**Supplementary Figure S1.** Data from GOBO database showed that SPIN1 expression was significantly highly expressed in basal-like or triple-negative breast cancer cells.

**
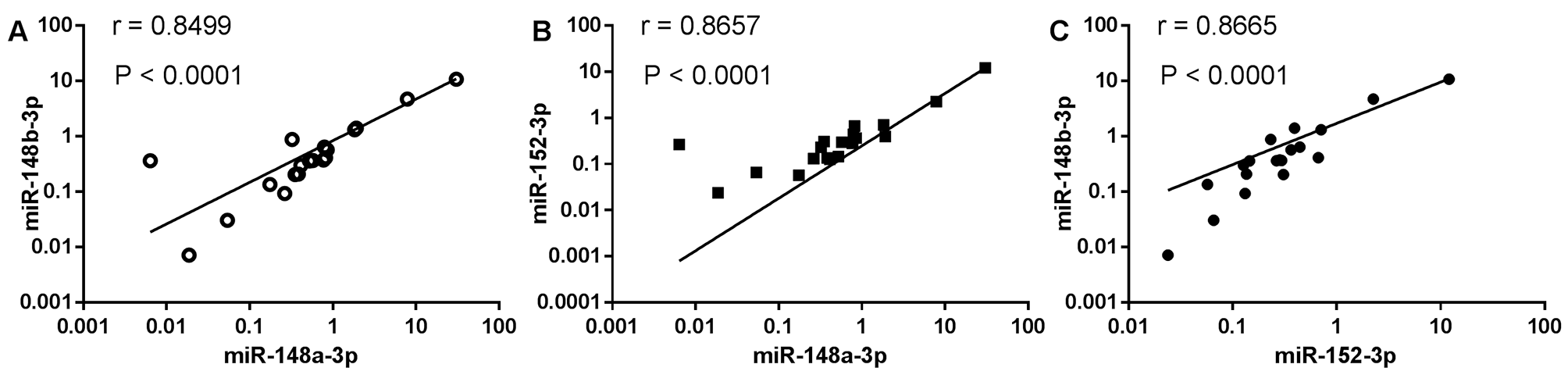
Supplementary Figure S2.** Expression of miR-148a-3p, miR-148b-3p or miR-152-3p was positively intercorrelated in MCF-7/ADM xenograft tumors (n = 20).
